# Impact of Calcium Sources on Soil Chemical Properties, Tomato Growth, Yield, and Quality

**DOI:** 10.1155/tswj/6653874

**Published:** 2025-06-22

**Authors:** Aruna Olasekan Adekiya, Timothy Oyebamiji Ogunbode, Vincent Ishola Esan, Olajire Adedokun, Iyabo Victoria Olatubi, Modupeola Hellen Ayegboyin

**Affiliations:** ^1^Agriculture Programme, College of Agriculture, Engineering and Science, Bowen University, Iwo, Nigeria; ^2^Pure and Applied Biology Programme, College of Agriculture, Engineering and Science, Bowen University, Iwo, Nigeria

**Keywords:** calcium nitrate, calcium sulfate, cow bone biochar, poultry manure, shelf life, tomato

## Abstract

Nigerian soils are generally light-textured and have a low cation exchange capacity, crucial for retaining exchangeable cations like calcium, which enhances tomato (*Solanum lycopersicum* L.) shelf life. Therefore, screenhouse studies were conducted to assess the effects of calcium fertilizers on soil chemical properties and tomato growth, yield, quality, and shelf life. Five Ca fertilizer sources were tested: no fertilizer, calcium sulfate, calcium nitrate, poultry manure, and cow bone biochar. Each was applied at 160 kg Ca ha^−1^ in a completely randomized design replicated three times. The soil used was classified as Alfisol. Results showed that applying different Ca sources improved soil chemical properties (organic matter, N, P, K, Ca, and Mg), plant growth (height and stem diameter), yield (fruit number and weight), and the mineral content of tomatoes. Results showed that calcium sulfate increased the Ca content of the soil by 1.25%, 9.82%, 20.11%, and 704% compared to calcium nitrate, poultry manure, biochar, and the control, respectively. Poultry manure enhances growth and yields the most due to its balanced nutrient supply. Poultry manure increased plant height by 10.1%, 11.2%, 20.3%, and 45.2% compared to calcium nitrate, calcium sulfate, biochar, and the control, respectively. Similarly, tomato fruit yield increased by 22.5%, 20.16%, 83.4%, and 382.5% relative to calcium nitrate, calcium sulfate, biochar, and the control, respectively. Relative to calcium nitrate, poultry manure, biochar, and control, calcium sulfate treatment reduced weight loss of tomato by 24.81%, 55.59%, 61.19%, and 104.99% and increased shelf life by 14.78%, 29.79%, 36.42%, and 69.44%, respectively. This effectiveness was attributed to its high Ca content. While poultry manure was effective in enhancing tomato yield and quality, it was not as effective as calcium sulfate in extending shelf life and reducing weight loss. Thus, future research should focus on the potential integration of poultry manure with calcium sulfate to develop an amendment that could improve yield, quality, and shelf life of tomatoes.

## 1. Introduction

Tomato (*Solanum lycopersicum* L.) stands as a pivotal vegetable crop with global cultivation, primarily sought after for its edible fruits [1]. Ranking as the third most crucial crop among vegetables [2], it holds significant agricultural importance. Nigeria, being the second-largest producer of tomato fruits in Africa and the thirteenth largest globally [3], plays a substantial role in the tomato industry. The usefulness of tomato fruits is evident in their various consumption forms, such as whole, in salads, soups, as juice, ketchup, paste, and puree [4]. Beyond their culinary appeal, these fruits offer a rich nutritional profile, containing essential vitamins (especially A and C), minerals, sugars, essential amino acids, iron, fiber, and phosphorus (P) [5]. Notably, lycopene is abundant in tomatoes, a carotenoid renowned for its antioxidant properties, contributing to the prevention of diseases such as cancer [6] and cardiovascular disorders [7].

In Nigeria, the increase in population growth and development has spurred an increased demand, consumption, and marketing of tomatoes. Despite this demand, tomato is a perishable vegetable crop with a shelf life of approximately 1 week at ambient temperature [8]. Consequently, various storage and preservation methods are employed to maintain the harvested fruits in an edible state for an extended period.

Tomato is typically harvested during periods of high moisture content and freshness [9]. The harvested fruit remains alive, undergoing respiration, until either consumed or subject to deterioration. Quality assessment is primarily based on factors such as color, texture, flavor, and nutritional value [10].

Postharvest losses of fresh fruits and vegetables, including tomatoes, are estimated to range from 20% to 50% in developing countries [11]. The storage of tomatoes is a crucial component of the postharvest handling chain. However, due to the high moisture content in the tissues of this crop, the act of storing becomes challenging [9]. Preserving vegetable products commercially in the tropics poses difficulties due to inadequate transportation networks and elevated environmental temperatures that promote decay rather than storage.

Several postharvest techniques have been employed to prolong the shelf life of tomatoes during storage, including the use of hydrogen sulfide [12], chitosan coating [13], abscisic acid [14], and essential oils [15]. Past research demonstrated that the application of calcium chloride (CaCl_2_) reduced fruit decay and enhanced tissue and cell wall hardness [16]. In a study by Shehata et al. [17], the impact of CaCl_2_, chitosan, hydrogen peroxide (H_2_O_2_), and ozonated water on the storage and quality of tomato (*Solanum lycopersicum* L. cv. 448) fruit stored at 10°C for 28 days was investigated. The results indicated that all the treatments significantly prolonged the shelf life and preserved the quality of tomato fruit compared to the control. Findings showed that chitosan and CaCl_2_ emerged as the most effective treatments for maintaining quality attributes, while CaCl_2_, abscisic acid, chitosan, H_2_O_2_, and ozonated water offer potential benefits in tomato postharvest storage; their use comes with significant drawbacks that cannot be overlooked. These include food safety concerns, environmental and health risks, potential negative impacts on sensory quality, and economic inefficiency. As a result, the application of calcium has garnered increased attention due to its capacity to delay the ripening of fruits and vegetables, along with extending their senescence and maintaining quality [18]. This underscores the potential for manipulating the shelf life of tomato through soil amendments. However, Nigerian soils are characterized by low cation exchange capacity (CEC), which is crucial for retaining exchangeable cations, including calcium [19]. Additionally, the majority of soils have a light texture and inadequate nutrient reserves, making them unsuitable for achieving high crop yields [20]. Sandy soils, in particular, exhibit low levels of organic matter and essential nutrients such as nitrogen (N), P, potassium (K), calcium, and magnesium. They also have a low CEC and a limited capacity to store and provide soil moisture [21]. Therefore, to enhance the yield, quality, and shelf life of tomatoes, the addition of calcium amendments is necessary.

Eboibi [22] conducted an experiment to assess the effects of eggshell-based manure (EBM), CaCl_2_, and their combined application in preventing tomato spoilage. Besides skin firmness, physiological weight loss, and spoilage rate, fruits treated with the combined treatment exhibited superior physical, mechanical, textural, and biochemical properties throughout the storage period. Studies on the manipulation of shelf life and qualities of tomato with the use of Ca-based soil amendment is not very common and therefore necessitating to be investigated. Most vegetables especially tomato is produced in Northern Nigeria where they are distributed over several kilometers to the south for sale. Calcium has been shown to influence the ripening process by reducing the sensitivity of fruit tissues to ethylene, the hormone responsible for ripening [23]. By moderating ethylene's effects, calcium can slow down the ripening process, allowing tomatoes to remain firm and fresh for a longer period postharvest [24]. This delayed ripening is particularly beneficial for extending shelf life, especially in supply chains that require longer storage or transportation times. Extending the shelf life of tomatoes through calcium-based soil amendments can lead to significant economic benefits for producers, distributors, and retailers. Reduced spoilage and waste translate to higher marketable yields, better prices, and increased profitability. Additionally, the enhanced quality of tomatoes can lead to greater consumer satisfaction and repeat purchases, further driving economic gains. The knowledge gap this study is aimed at addressing includes the following key areas: While various postharvest treatments such as CaCl_2_, chitosan, and essential oils have been explored for extending tomato shelf life, fewer studies have investigated the role of soil-applied calcium amendments in influencing postharvest quality and storability. Again, Nigerian soils, particularly sandy soils, are characterized by low CEC and inadequate nutrient reserves, including calcium. This limits their ability to support high tomato yields and maintain fruit quality. Research on the impact of soil-based calcium amendments in such conditions is scarce. Furthermore, most studies treat soil fertility and postharvest management as separate areas. This research bridges that gap by assessing how calcium-based fertilizers influence both soil properties and postharvest tomato shelf life. Therefore, the objective of this study was to assess the effects of calcium-based fertilizers on soil chemical properties, growth, yield, quality, and shelf life of tomato. It was hypothesized that soil chemical properties, growth, yield, quality, and shelf life of tomato will respond to calcium-based fertilizers differently; therefore, experiments were conducted to validate this hypothesis.

## 2. Materials and Methods

In 2024, two simultaneous experiments were conducted at Sites A and B within the same screenhouse between February and May at the Teaching and Research Farm of Bowen University, Iwo, Osun State, Nigeria. Site B was used to verify findings from site A. The University is located at 7.6236°N, 4.1890°E, with an elevation of 312 m. The screenhouse has a galvanized iron frame, UV protection covering, insect-resistant netting, and a granite floor. Temperature and humidity inside averaged 31°C and 75%, respectively. The soil in Iwo is classified as Oxic Haplustalf (USDA) or Luvisol [25].

### 2.1. Treatment Application and Preparation of Soil Amendment

The experiment included five calcium fertilizer sources: no fertilizer, calcium sulfate, calcium nitrate, poultry manure (PM), and cow bone biochar, applied at 160 kg Ca ha^−1^. The treatments were arranged in a completely randomized design (CRD) with three replications.

Cow bone biochar was produced by pyrolyzing bones from an Iwo abattoir at ~500°C in a sealed metal drum, cooled, ground, and sieved [26]. Calcium sulfate and calcium nitrate were purchased from an agrochemical store in Ibadan, while PM was obtained from Bowen University's Poultry Unit.

Soil samples (0–15 cm depth) were collected, mixed, sieved (2 mm), and placed into perforated grow bags (15-kg soil each). Each treatment had four grow bags, with 20 bags per replicate at Site A. An identical set was placed adjacent within the screenhouse for Site B.

### 2.2. Application of Soil Amendments

The fertilizers were applied based on 160 kg Ca/ha [27]. The Ca in the calcium sulfate, calcium nitrate, PM and cow bone biochar was 29.4%, 24.39%, 6.08%, and 5.0%, respectively:

(1) $$\begin{array}{c} \text{calcium sulfate}=\left(\frac{160\;}{29.4}\right)\times 100=544.2\text{ kg }{\text{ha}}^{-1}. \end{array}$$

But 1 ha of land contains 2,000,000 kg of soil as standard [28]; it then means 1 ha = 2,000,000 kg of soil = 544.2 kg calcium sulfate.

Therefore,

(2) $$\begin{array}{c} 15\text{ kg soil}=\left(\frac{544.2\;}{2,000,000}\right)\times 15=0.00408\text{ kg or }4.08\text{ g calcium sulfate}. \end{array}$$

Following the above, calcium sulfate was applied at 544.2 kg ha^−1^ and 4.08 g in a 15-kg grow bag, calcium nitrate was applied at 655.7 kg ha^−1^ and 4.9 g in a 15-kg grow bag, PM was applied at 2666.6 kg ha^−1^ and 19.9 g in a 15-kg grow bag, and cow bone biochar was applied at 3200 kg ha^−1^ and 24.0 g in a 15-kg grow bag. The control receives no amendment. Cow bone biochar was incorporated into the soil using a hand trowel and allowed to mineralize for 4 weeks before transplanting tomatoes into the grow bags. PM treatment was incorporated into the soil 1 week before transplanting tomatoes. Watering was done immediately and continued every other day till the day of transplanting. Calcium sulfate and calcium nitrate treatments were applied 5 days after transplanting.

### 2.3. Nursery Establishment and Transplanting

Seeds of Ife Local, an indigenous tomato variety, were sown in loamy soil-filled seed trays in the screenhouse. Watering was done daily to field capacity. After 3 weeks, seedlings were transplanted in the evening with a ball of earth to minimize root damage, one plant per grow bag. Spacing was 60 cm between bags and 90 cm between rows. Each site had 60 plants. Watering continued to field capacity throughout the experiment, which lasted 100 days. Weeding was done manually.

### 2.4. Chemical Analyses of Soil, Biochar, and PM

Pretreatment soil samples were air-dried, sieved (2 mm), and analyzed. Particle size distribution was determined by the hydrometer method [29]. Organic carbon (OC) was measured using the Walkley–Black method [30], total N by micro-Kjeldahl digestion [31], and available P by Bray-1 extraction [32]. Exchangeable cations (K, Na, Ca, and Mg) were extracted using 1 M ammonium acetate (NH_4_OAc) at pH 7 [33]. The soil-to-solution ratio was 1:10 (*w*/*v*), and the mixture was shaken for 30 min before filtration. The filtrate was then analyzed for cation concentrations using atomic absorption spectrophotometry (AAS) Postexperiment soil samples were analyzed similarly. Biochar and PM were analyzed for OC, N, P, K, Ca, Na, and Mg [34].

### 2.5. Determination of Growth and Yield Components

Three plants per treatment were assessed for plant height, stem diameter, and leaf number at midflowering (~28 days after transplanting). Plant height was measured with a meter rule, leaves were manually counted, and stem diameter was measured with a vernier caliper. Tomato fruit weight was evaluated based on the cumulative weight of harvests per grow bag.

### 2.6. Shelf-Life Determination

The first set of tomato fruits harvested was cleaned with a clean cloth; after which they were sorted into different treatment groups, with each group containing five fruits and three replicates. They were then properly arranged on a clean table in the laboratory to determine their shelf lives.

Parameters assessed include the following:

i. Weight loss: The weight of each tomato fruit was taken soon after harvesting and during each experimental day using a weighing balance. The total weight loss was calculated by taking the difference between the initial and final weight (at 5-day interval till full deterioration) of the fruits during the storage period using the formula:

 $$\begin{array}{c} \text{WL }\left(\%\right)=\left(\frac{\text{initial weight}-\text{final weight }}{\text{initial weight}}\right)\times 100. \end{array}$$

WL is the weight loss.

ii. Shelf life: This was carried out by counting the number of days from the day of harvesting the tomato fruit to the day it was considered bad and below marketable condition. That is, the tomato exhibits significant shriveling, discoloration, decay, excessive softness, mushiness, signs of collapse, or emits an unpleasant smell, all of which affect its visual appeal, firmness, and marketability.

### 2.7. Mineral and Proximate Composition

Mature tomato fruits of uniform size were analyzed for mineral content (Cu, Fe, Mg, K, Ca, and Na) following AOAC [34]. One gram of fruit was digested in HNO_3_, H_2_SO_4_, and HClO_4_ (7 : 2 : 1 *v*/*v*), and mineral content was measured using atomic absorption spectroscopy.

### 2.8. Data Analysis

Data were analyzed using ANOVA in SPSS [35]. Treatment means were compared using Duncan's multiple range test (DMRT) at a 5% probability level.

## 3. Results

### 3.1. Soil Properties Prior to Experimentation and Analysis of Soil Amendments Used

Tables 1 and 2, respectively, show the results of the soil potted in grow bags before the addition of amendments and the chemical analysis of soil amendments used. The particle size analysis indicated that the soil was sandy loam in texture with high sand values and low values of both silt and clay. The soil was low in organic matter (2.04%), total N (0.10%), available P (4.99 mg kg^−1^), Ca (1.70 cmol kg^−1^), and Mg (0.30 cmol kg^−1^) but adequate in exchangeable K (0.19 cmol kg^−1^) (Table 1) according to the critical levels of 3.0% organic matter, 0.20% N, 10.0 mg/kg available P, 0.16–0.20 cmol/kg K, 2.0 cmol/kg exchangeable Ca, and 0.40 cmol/kg exchangeable Mg recommended for crop production in ecological zones of Nigeria [36]. Biochar has higher values of OC, P, and Mg relative to PM, whereas PM has higher values of N, K, and Na relative to biochar (Table 2). The increasing order of Ca among the soil amendments was biochar < PM < calcium nitrate < calcium sulfate (Table 2).

### 3.2. Effects of Various Ca Sources on Soil Chemical Properties

Results of the various Ca soil amendments on soil chemical properties are presented in Table 3. All Ca sources of soil amendment (PM, biochar, calcium sulfate, and calcium nitrate) increased soil chemical properties relative to the control. Biochar treatment at both sites increased P and Mg values relative to other amendments. PM increased soil organic matter (SOM) and K contents of the soil relative to other treatments. Calcium sulfate and calcium nitrate treatments increased N and Ca levels of the soil relative to PM, biochar, and the control. There were no significant differences (*p* < 0.05) between calcium sulfate and calcium nitrate treatments for SOM, N, K, Ca, and Mg. Using the average from the two sites, calcium sulfate had the highest soil calcium content (12.96 cmol/kg), which was not significantly different (*p* < 0.05) from that of calcium nitrate (12.80 cmol/kg). This was followed by PM (11.81 cmol/kg) and biochar (10.79 cmol/kg), while the lowest value was observed in the control (1.61 cmol/kg). These results showed that calcium sulfate increased the Ca content of the soil by 1.25%, 9.82%, 20.11%, and 704% compared to calcium nitrate, PM, biochar, and the control, respectively.

### 3.3. Effects of Various Ca Sources on Growth and Yield of Tomato

Results of the various Ca amendments on growth and yield of tomato are presented in Table 4. The different Ca sources increased growth (plant height, stem diameter, and number of leaves) and yield (number of fruits and fruit weights) parameters of tomato relative to the control. PM treatment increased growth and yield parameters of tomato relative to other Ca sources. There were no significant differences (*p* < 0.05) in plant height, stem diameter, number of leaves, number of fruits, and fruit weight values between calcium sulfate and calcium nitrate. Using the average from the two sites, PM had the highest tomato yield (2485.6 g), followed by calcium sulfate (2069.7 g), which was not significantly different (*p* < 0.05) from calcium nitrate (2029.5 g). Biochar resulted in a lower yield (1236.5 g), while the lowest yield was observed in the control (533.9 g). PM increased plant height by 10.1%, 11.2%, 20.3%, and 45.2% compared to calcium nitrate, calcium sulfate, biochar, and the control, respectively. Similarly, tomato fruit yield increased by 22.5%, 20.16%, 83.4%, and 382.5% relative to calcium nitrate, calcium sulfate, biochar, and the control, respectively.

### 3.4. Effects of Various Ca Sources on Mineral Contents of Tomato Fruits

Results showing the effects of various Ca sources of soil amendments on the mineral contents of tomato fruits are presented in Table 5. The application of amendments increased the mineral (Na, Cu, Fe, Ca, Zn, and Mg) concentrations of tomato fruits relative to the control. Except for Ca, PM significantly increased the mineral contents of tomato relative to other amendments. Calcium sulfate significantly increased Ca concentration relative to others. There were no significant differences (*p* < 0.05) between calcium sulfate and calcium nitrate for Cu and Fe.

### 3.5. Effects of Various Ca Sources on Shelf Life

**Figure 1:**
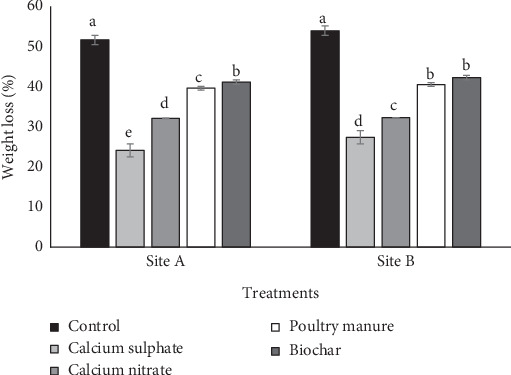
Effect of various Ca sources on weight loss of tomato. Note: Vertical bars show standard error of paired comparisons; bars marked with different letters show means significantly different at 5% level using Duncan's multiple range test.

**Figure 2:**
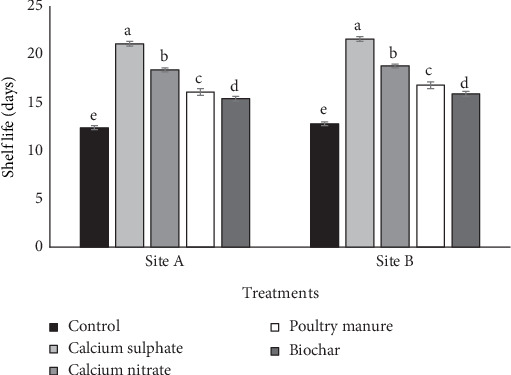
Effect of various Ca sources on shelf life of tomato. Note: Vertical bars show standard error of paired comparisons; bars marked with different letters show means significantly different at 5% level using Duncan's multiple range test.

The results showing the effects of various Ca sources on weight loss and shelf life of tomato are showed in Figures 1 and 2, respectively. Different sources of Ca reduced tomato fruit weight loss relative to no application (control). At both sites, among different Ca sources, calcium sulfate fertilizer treatment reduced weight loss less while biochar reduced the most. Using the average from the two sites and relative to calcium nitrate, PM, biochar, and control, calcium sulfate treatment reduced weight loss by 24.81%, 55.59%, 61.19%, and 104.99%, respectively. The application of the amendment increased the shelf life of tomato relative to the control. Using average values from the two sites and relative to calcium nitrate, PM, biochar, and control, calcium sulfate fertilizer increased the shelf life of tomato by 14.78%, 29.79%, 36.42%, and 69.44%, respectively.

## 4. Discussion

The increase in N, P, K, Ca, and Mg contents of the soil due to the addition of amendments could be due to the presence of nutrients in the amendments (Table 2). PM contains organic matter which releases nutrients into the soil during decomposition. Studies [37, 38] have shown that PM increased soil OM, N, P, and CEC, and this was attributed to the availability and adequate supply of organic matter. When applied to soil, it increases the availability of these nutrients, leading to improved soil fertility. The organic matter in PM enhances soil structure, increases water retention, and promotes microbial activity, which further improves nutrient cycling and availability.

Biochar is a stable form of carbon which, when added to soil, increases its carbon content. This enhances soil fertility by improving nutrient retention and CEC, making nutrients more available to plants [26, 39, 40]. Biochar buffers soil pH, making it more favorable for nutrient availability and plant uptake [23].

Calcium sulfate and calcium nitrate are a direct source of calcium, which increases the calcium content in the soil. They improve soil structure by promoting the aggregation of soil particles. Better soil structure reduces soil compaction and improves aeration, leading to enhanced microbial activity. Microorganisms play a key role in the mineralization of organic matter, converting it into forms of N, P, K, and magnesium that are available to plants [41]. Calcium nitrate is primarily a source of N in the form of nitrate (NO_3_^−^), which is readily available for plant uptake. This directly increases the N content in the soil.

Biochar soil has higher P content due to the fact that cow bone contains a lot of P relative to PM and calcium sulfate and calcium nitrate with no P at all (Table 2). Again, biochar-treated soil has higher P relative to other treatments due to the fact that biochar application reduced P leaching, resulting in significantly higher P content in the soil compared to the control, thereby maintaining a reasonable level of P in the soil [42]. The noticeable increase in Mg in soils treated with biochar can also be attributed to the presence of cation exchange sites on the biochar's surface [43].

The decreasing order of tomato yield was PM > calcium sulfate = calcium nitrate > biochar > control. All the amendments increased growth and tomato yield relative to the control. The positive effect of the Ca amendments on growth and tomato yield could be due to the contribution made by the amendments to fertility status of the soils as the soils were initially low in nutrient content. PM when decomposed releases both macro and micronutrients as well as enhances the physiochemical properties of the soil for improvement of tomato growth.

The calcium in calcium sulfate and calcium nitrate improves soil structure by helping to break up compacted soils [44]. This enhances root penetration, allowing tomato plants to access water and nutrients more effectively. Better root development leads to improved plant vigor and productivity [45]. In addition, the N in calcium nitrate is in the form of nitrate, which is readily available for plant uptake. This form of N promotes rapid growth and development, leading to lush foliage and increased photosynthesis, which supports higher tomato fruit production.

Biochar improves soil structure by increasing porosity and aeration [46]. This helps in better root penetration and water retention, ensuring that tomato plants have access to the water they need, even during dry periods. Improved soil structure also enhances the movement of air and nutrients within the soil. The porous structure of biochar provides an excellent habitat for beneficial soil microorganisms, enhancing their activity and proliferation in the soil [26]. These microbes play a crucial role in breaking down organic matter, cycling nutrients, and protecting plants from pathogens. Enhanced microbial activity leads to a healthier soil ecosystem, which in turn supports better tomato growth and higher yields. Cow bone biochar is a rich source of P and calcium (Table 2), two essential nutrients for tomato plants [47]. P is vital for root development, flowering, and fruiting [48], while calcium strengthens cell walls [49], preventing disorders like blossom-end rot [50]. These nutrients are slowly released from the biochar, ensuring a steady supply for the plants.

Application of PM might have led to a better yield of tomatoes compared to cow bone biochar, calcium sulfate, and calcium nitrate due to the fact that PM provides a more balanced mix of nutrients, including N, P, K, and trace elements, which are essential for the overall growth of tomatoes (Table 2). This contrasts with calcium sulfate and calcium nitrate, which primarily supply specific nutrients like calcium and N. The more comprehensive nutrient profile in PM supports various stages of tomato growth, from vegetative development to fruiting. In addition, PM is rich in organic matter, which improves soil structure, water retention, and aeration [51]. This organic matter also enhances microbial activity in the soil [52], contributing to better nutrient availability and healthier root systems. Cow bone biochar, though beneficial, does not supply the same level of organic matter, focusing more on P and calcium content. Also, the nutrients in PM are released slowly as the organic matter decomposes, providing a steady nutrient supply over time. This reduces the risk of nutrient leaching and ensures that tomatoes receive a continuous supply of nutrients throughout the growing season, leading to more consistent growth and higher yields. In contrast, fertilizers like calcium nitrate might provide a quick boost of nutrients but could also lead to rapid nutrient loss through leaching or imbalances. Furthermore, the organic matter in PM supports a diverse and active soil microbial community [53]. These microbes help decompose organic matter, making more nutrients available to plants and improving soil health. Although biochar can enhance microbial habitats, it does not provide the same level of readily available organic matter as PM.

The fact that Ca fertilizers increased tomato mineral contents compared with the control could be attributed to increased availability of the nutrients in soil as a result of the mineralization, leading to increased uptake by the tomato plants. PM has the highest values of Na, Cu, Fe, Zn, and Mg in tomato fruits. This might be due to differences in the chemical composition of PM compared to calcium sulfate, calcium nitrate, and biochar and its positive effect on soil ecology and plant metabolism [54]. Calcium sulfate contains Ca and S only, and calcium nitrate contains Ca and N only, although biochar contains nutrient like PM but of inferior quality due to high C:N ratio (43.43) which make them not to be available/mineralized faster for absorption by tomato plant. PM has low C:N ratio (4.24) and contains a wide range of essential nutrients, including macronutrients like N, P, and K, as well as micronutrients such as magnesium, sulfur, copper, zinc, and manganese. This balanced nutrient composition supports not only tomato growth but also the development of nutrient-rich tomato fruits. It appears logical that the composition and availability of mineral nutrients in the soil significantly influence the quantity and quality of nutrients taken up by plants. For instance, studies have shown that plants cultivated in organic farming systems tend to exhibit higher levels of micronutrients compared to those grown under conventional farming practices [55].

PM, cow bone biochar, calcium sulfate, and calcium nitrate fertilizers reduced weight loss and increased the shelf life of tomato relative to the control (unamended soil) due to the fact that application of these amendments improved fruit quality by enhancing nutrient uptake through a balanced supply of nutrients, including N, P, K, and micronutrients, all of which improved the overall health and strength of the tomato plants. Healthier plants produce fruits with stronger cell walls and better structural integrity, which helps to reduce weight loss during storage. Again, these amendments are excellent sources of calcium (Table 2), which is critical for cell wall strength and integrity. Calcium helps maintain the firmness of tomatoes, reducing water loss and extending the shelf life. This is due to the fact that calcium is a vital component of the plant cell wall, where it binds with pectins to form calcium pectate [56]. This compound is a critical part of the middle lamella, which is the layer that cements neighboring plant cells together [57]. Stronger cell walls help maintain the structural integrity of the tomato fruit [58], making it less prone to mechanical damage, water loss, and microbial invasion. It has been reported that strawberry uptake of exogenous calcium ions increases the amount of chelate-soluble pectins, thus enhancing cell wall stability and preventing the dissolution of the middle lamella [59, 60]. In addition, calcium stabilizes cell membranes by interacting with phospholipids, reducing the permeability of the cell membrane. This stabilization helps to maintain cellular structure and function, reducing the rate of water loss (transpiration) from the fruit during storage [61]. Lower water loss translates to less weight loss and longer shelf life. Besides, calcium has been shown to slow down the production of ethylene, a plant hormone that promotes ripening and senescence (aging) [62]. By delaying ethylene production, calcium helps to slow down the ripening process, thereby extending the shelf life of tomatoes. Slower ripening reduces the softening and overripening of fruits, which are major contributors to weight loss and spoilage. In newly harvested New Queen melons treated with CaCl_2_ and 1-methylcyclopropene (1-MCP) alone or in combination before storage [63], respiration rate, ethylene release, and the activity and gene expression of pectinases such as polygalacturonase (PG), pectin methylesterase (PME), and pectate lyase (PL) were dramatically decreased by treatments with 0.18 mol/L CaCl_2_ and/or 1 *μ*L/L 1-MCP. It has also been reported that calcium can preserve fruit firmness by slowing down the ripening and aging process, improving cold storage resistance by regulating reactive oxygen species levels, inhibiting postharvest diseases, and maintaining overall fruit quality [64–66]. Furthermore, calcium inhibits the activity of enzymes like PG and PME [67], which break down pectin in the cell walls during ripening. By inhibiting these enzymes, calcium helps to maintain the structural integrity of cell walls, delaying softening and reducing the rate of degradation in storage. In an experiment conducted to evaluate the effects of EBM, CaCl_2_, and their combined application in preventing spoilage of tomato fruits [22], it was observed that tomato fruits produced with CaCl_2_ experienced the highest skin firmness, lowest physiological weight loss, and minimum spoilage; fruits produced with EBM exhibited the maximum physiological weight loss, highest spoilage rate, and minimum skin firmness.

In this experiment, calcium sulfate has the longest shelf life and reduced weight loss in storage; this was a result of the Ca content of the various Ca sources used as amendment. The higher the calcium content, the longer the shelf life and the greater the reduction in weight loss. In both sites' average, the correlation coefficient between Ca contents of amendment used (PM, calcium sulfate, calcium nitrate, and biochar) and tomato fruit weight loss and shelf life was all significant with *R* values of −0.979 and 0.953, respectively, at *p* < 0.05.

PM is a valuable organic amendment that enhances soil fertility by supplying nutrients and organic matter [37, 51]. However, improper management can lead to nutrient leaching, particularly of N and P, which may contaminate water bodies and cause environmental issues [68]. Both calcium sulfate and calcium nitrate are environmentally stable soil amendments that provide essential nutrients like calcium and sulfur or N. However, they lack the organic matter and soil biological benefits that manure contributes, limiting their role in improving long-term soil health.

## 5. How Farmers Can Economically Integrate These Findings Into Their Practices

The findings offer valuable insights for tomato farmers looking to improve yield, quality, and shelf life while maintaining economic efficiency. Practical implications include the following.

Farmers can economically integrate PM with calcium sulfate in their fertilizer management. PM can be applied at planting to enhance soil fertility, plant growth, and fruit yield. Calcium sulfate, on the other hand, can be applied later in the growing cycle or during fruit development to improve calcium availability, enhancing shelf life and reducing weight loss.

For smallholder farmers, sourcing PM locally reduces costs, while calcium sulfate can be applied in smaller quantities due to its targeted effect. This minimizes expenses while maximizing the benefits of both amendments. The combination of PM and calcium sulfate ensures not only higher yields and better-quality tomatoes but also longer shelf life and reduced weight loss, which are critical for accessing distant markets and reducing postharvest losses.

## 6. Conclusion

This study demonstrated that the application of different calcium sources significantly influenced soil chemical properties, growth, yield, mineral content, and shelf life of tomatoes. Among the amendments evaluated, PM proved to be the most effective in improving soil fertility, enhancing plant growth, and increasing tomato yield due to its high organic matter and nutrient content. PM increased yield by 22.5%, 20.16%, 83.4%, and 382.5% compared to calcium nitrate, calcium sulfate, biochar, and the control, respectively. Calcium sulfate and calcium nitrate also contributed significantly to soil calcium levels, with calcium sulfate emerging as the most effective in extending the shelf life of harvested tomatoes and minimizing postharvest weight loss. Relative to calcium nitrate, PM, biochar, and control, calcium sulfate treatment reduced weight loss of tomato by 24.81%, 55.59%, 61.19%, and 104.99% and increased shelf life by 14.78%, 29.79%, 36.42%, and 69.44%, respectively. Further research should explore integrating PM and calcium sulfate to maximize both yield and storage quality.

## Figures and Tables

**Table 1:** Initial physical and chemical properties of the soil.

**Property**	**Value**
Sand (%)	78.0
Silt (%)	10.0
Clay (%)	12.0
Textural class	Sandy loam
Organic matter (%)	2.04
pH (water)	6.05
N (%)	0.10
P (mg kg^−1^)	4.99
K (cmol kg^−1^)	0.19
Ca (cmol kg^−1^)	1.70
Na (cmol kg^−1^)	0.26
Mg (cmol kg^−1^)	0.30

**Table 2:** Chemical properties of various Ca sources.

	**Organic carbon (%)**	**N (%)**	**C:N ratio**	**P (%)**	**K (%)**	**Na (%)**	**Ca (%)**	**Mg (%)**
Calcium sulfate	NA	NA	NA	NA	NA	NA	29.4	NA
Calcium nitrate	NA	NA	NA	NA	NA	NA	24.39	NA
Poultry manure	18.40	1.91	4.24	0.49	1.14	0.68	6.08	2.72
Cow bone biochar	59.50	1.37	43.43	0.51	0.92	0.61	5.00	2.60

Abbreviation: NA, not applicable.

**Table 3:** Effect of various Ca sources on soil chemical properties.

	**SOM (%)**		**N (%)**		**P (mg kg** ^**−1**^ **)**		**K (cmol kg** ^**−1**^ **)**		**Ca (cmol kg** ^**−1**^ **)**		**Mg (cmol kg** ^**−1**^ **)**	
	**Site A**	**Site B**	**Site A**	**Site B**	**Site A**	**Site B**	**Site A**	**Site B**	**Site A**	**Site B**	**Site A**	**Site B**
Control	2.00d	1.98d	0.09d	0.09d	4.55e	4.61e	0.17d	0.17d	1.56d	1.66d	0.29d	0.28d
Calcium sulfate	2.29c	2.22c	0.25a	0.25a	13.22d	13.45d	1.58c	1.60c	12.95a	12.98a	6.00c	6.10b
Calcium nitrate	2.21c	2.30c	0.23a	0.24a	13.64c	13.86c	1.63c	1.64c	12.75a	12.85a	6.10c	6.72b
Poultry manure	3.26a	3.28a	0.21b	0.23b	26.33b	25.83b	1.93a	1.95a	11.73b	11.81b	7.13b	7.18a
Biochar	2.66b	2.68b	0.11c	0.12c	29.41a	29.81a	1.83b	1.86b	10.73c	10.85c	8.86a	8.91a
SE	0.22	0.23	0.032	0.033	4.58	4.56	0.32	0.33	2.13	2.13	1.44	1.46

*Note:* Values followed by similar letters under the same column are not significantly different at *p* ≤ 0.05 according to Duncan's multiple range test.

Abbreviation:SOM, soil organic matter.

**Table 4:** Effect of various Ca sources on growth and yield of tomato.

	**Plant height (cm)**		**Stem diameter (cm)**		**Number of leaves**		**Number of fruits/plants**		**Fruit weight/plant (g)**	
	**Site A**	**Site B**	**Site A**	**Site B**	**Site A**	**Site B**	**Site A**	**Site B**	**Site A**	**Site B**
Control	58.7d	59.2d	2.1d	2.3d	8.5d	8.5c	9.1d	9.4d	541.1d	526.8d
Calcium sulfate	77.6b	77.9b	3.6b	3.8b	9.1b	9.2b	13.6b	13.8b	2010.8b	2128.6b
Calcium nitrate	76.4b	77.6b	3.5b	3.6b	9.2ab	9.5b	13.0b	13.6b	1990.6b	2068.4b
Poultry manure	84.1a	87.1a	4.1a	3.9a	10.0a	11.0a	15.1a	15.4a	2480.3a	2491.8a
Biochar	70.8c	71.5c	3.0c	3.1c	8.8 cd	8.7bc	12.6c	12.8c	1310.6c	1401.5c
SE	4.27	4.60	0.34	0.29	0.25	0.44	0.99	0.99	337.56	347.06

*Note:* Values followed by similar letters under the same column are not significantly different at *p* ≤ 0.05 according to Duncan's multiple range test.

**Table 5:** Effect of various Ca sources on mineral contents of tomato.

	**Na (mg kg** ^**−1**^ **)**		**Cu (mg kg** ^**−1**^ **)**		**Fe (mg kg** ^**−1**^ **)**		**Ca (mg kg** ^**−1**^ **)**		**Zn (mg kg** ^**−1**^ **)**		**Mg (mg kg** ^**−1**^ **)**	
	**Site A**	**Site B**	**Site A**	**Site B**	**Site A**	**Site B**	**Site A**	**Site B**	**Site A**	**Site B**	**Site A**	**Site B**
Control	1435.1e	1411.4e	8.88d	8.89d	45.01d	46.81d	2014.1e	2281.1e	15.18e	14.89e	444.71e	434.81e
Calcium sulfate	1968.2c	1869.2	21.76b	22.43b	75.07b	75.91b	5100.04a	5124.10a	40.57b	40.47b	1018.1c	1112.78c
Calcium nitrate	2442.3b	2391.4b	20.25b	21.68b	70.07b	71.09b	4831.08b	4848.2b	31.84c	32.86c	978.64d	985.98d
Poultry manure	3642.1a	3553.2a	26.68a	26.98a	80.93a	80.11a	3022.1d	3044.1d	45.26a	44.89a	2312.4a	2324.8a
Biochar	1603.8d	16554.1d	9.32c	9.89c	55.71c	56.94c	4521.2c	4642.1c	18.19d	19.22d	1669.1b	1689.8b
SE	395.66	2871.87	3.54	3.62	6.58	6.21	592.46	559.59	5.84	5.84	322.04	322.79

*Note:* Values followed by similar letters under the same column are not significantly different at *p* ≤ 0.05 according to Duncan's multiple range test.

## Data Availability

All data used are included within the article.
